# Unusual presentation of complicated relapsing fever with spontaneous hemoperitoneum mimicking surgical acute abdomen: a case report

**DOI:** 10.1186/s12245-025-01035-7

**Published:** 2025-10-17

**Authors:** Yemane Gebremedhin Tesfay, Habtamu Kebede Adera, Zelalem Getahun Demissie, Dirijit Mamo Alemu, Menbeu Sultan Mohammed, Getaw Worku Hassen, Mohammed Kalifa Nuguse

**Affiliations:** 1 Department of Emergency and Critical Care Medicine, St. Paul’s Hospital Millennium Medical College, Addis Ababa, Ethiopia; 2 Department of Emergency and Critical Care Medicine, Wollega University Institute of Health Sciences, Nekemte, Ethiopia; 3 Department of Intensive Care Medicine, St. Paul’s Hospital Millennium Medical College, Addis Ababa, Ethiopia; 4 Emergency Medicine at New York Medical College, Metropolitan Hospital Center, New York, USA

**Keywords:** Unusual presentation, Relapsing fever, Hemoperitoneum, Acute abdomen, Case report

## Abstract

**Background:**

Louse-borne relapsing fever (LBRF) is a prevalent disease in Ethiopia, affecting malnourished and impoverished populations. Historically fatal, mortality has decreased to less than 5% with antibiotics. Symptoms include high fever, rigors, myalgia, hepatosplenomegaly, jaundice, and petechial rash. Diagnosis is challenging due to Giemsa-stained blood films and PCR in resource-limited settings.

**Case Presentation:**

A 29-year-old Ethiopian patient experienced abdominal pain, vomiting, diarrhea, and high fever for 3 days. Physical examination revealed low blood pressure, oxygen saturation, tachycardia, decreased air entry, and a tender abdomen. A bedside ultrasound revealed bilateral pleural collection, dense B lines, an air bronchogram, and abdominal collection. The patient had thrombocytopenia, leukocytosis, acute kidney injury, elevated transaminase levels, and Borrelia spirochetes in her blood. The patient recovered fully within 8 days after respiratory failure.

**Discussion:**

This case highlights the importance of recognizing relapsing fever (RF) as a critical mimic of surgical abdomen, preventing unnecessary surgical interventions in hemorrhagic abdominal emergencies.

**Conclusion:**

A life-threatening Borrelia-induced hemoperitoneum in Ethiopia was successfully managed, despite complications of surgical acute abdomen with hemoperitoneum, severe thrombocytopenia, and multiorgan involvement, highlighting the importance of infectious consideration in acute abdomen.

## Introduction

Louse-borne relapsing fever (LBRF), caused by Borrelia recurrentis, remains endemic in Ethiopia and disproportionately affects malnourished and impoverished populations during seasonal epidemics [1].

Historically fatal in 30–70% of untreated cases, mortality has decreased to < 5% with prompt antibiotics, although treatment is complicated by universal Jarisch–Herxheimer reactions (J-HRs) [1, 2].

Classic louse-borne relapsing (LBRF) presents with high fever, rigors, and myalgia, accompanied by hepatosplenomegaly (50–77%), jaundice (7–70%), and petechial rash (2–80%) [3, 4].

Severe complications include myocarditis, acute respiratory distress syndrome (ARDS), cerebral hemorrhage, coagulopathy, thrombocytopenia, and splenic rupture [1, 5].

Diagnosis in endemic areas relies on Giemsa-stained blood films with a sensitivity of less than 5% during afebrile periods, [6] as polymerase chain reaction (PCR) remains inaccessible in resource-limited settings despite 100% specificity [7].

This case report highlights diagnostic challenges in diagnosing LBRF-associated hemoperitoneum, with a focus on zoonotic infections in acute abdominal emergencies, especially in febrile, thrombocytopenic patients from endemic regions.

## Case presentation

A 29-year-old male patient from Addis-Ababa, Ethiopia, reported experiencing non-radiating lower abdominal pain for three days, accompanied by vomiting, diarrhea, fever, chills, and headache. He also had a productive cough with blood-tinged sputum and shortness of breath the day before his emergency visit to Saint Paul’s Hospital Millennium Medical College’s (SPHMMC) Emergency Department (ED) on December 11, 2024.

The patient has no allergies to medications, foods, or environmental substances and is not currently taking any recreational drugs or stimulants. He has persistent vomiting, is unable to tolerate solid or liquid intake for more than 12 h, and has a 5-year smoking habit. He has no travel history to malaria endemic areas, and there is no report of dengue fever in Addis-Ababa.

The individual was imprisoned for two days before his current illness and was reported to have louse infestation at his initial visit to the prison clinic, and was deloused thoroughly. After being given 1000 ml of 0.9% NaCl, the patient was referred to our hospital for an acute abdomen secondary to peritonitis secondary to a perforated viscus, requiring possible surgical intervention.

Upon arrival at the ED, the patient had a patent and protected airway but was in respiratory distress at 35 breaths/minute, 75% oxygen saturation, and decreased air entry. After receiving 15 L/min of oxygen with a non-rebreather face mask, the saturation improved to 94%, but the work of breathing and respiratory rate remained unchanged.

His extremities were cold, with a pulse rate (PR) of 120/minute and feeble. Blood pressure (BP) was 70/50 mmHg, and a normal cardiac examination was performed. Two large-bore intravenous (IV) lines were opened. He was resuscitated with normal saline and given the first dose of cefepime and vancomycin. Despite this, there was no improvement in BP, and noradrenaline infusion was initiated. His random blood glucose level was 200 mg/dl, and he was conscious and oriented.

Bedside ultrasound revealed bilateral pleural fluid collection, more on the right side, diffuse B lines bilaterally with consolidation and air bronchogram. It also revealed significant peritoneal and pelvic collections with a collapsing inferior vena cava (IVC).

Then, his BP became 100/60 mm Hg, his PR was 87/minute, his RR was 30–40/minute, his SpO_2_ was 87% with 15 L/min of oxygen with a nonrebreather face mask (NRB), and his temperature was 38.7 °C.

The abdomen was relatively soft despite the presence of direct and rebound tenderness throughout the abdomen.

While resuscitation continued, the surgical team was consulted immediately, suggested performing a diagnostic peritoneal tap, which revealed frank blood, and decided to continue with nonsurgical management given the patient’s hemodynamic instability and lack of focal peritonitis.

**Fig. 1:**
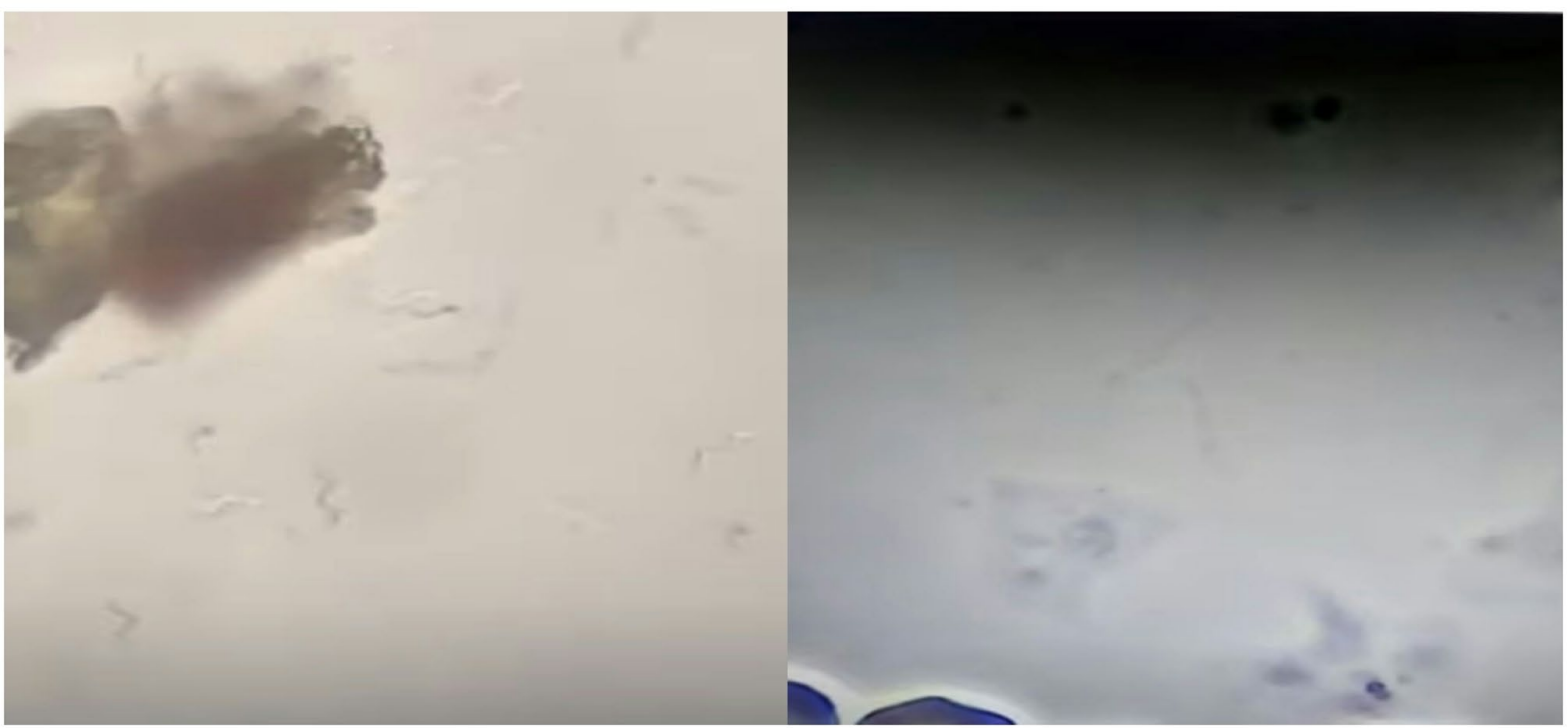
Peripheral blood smear showing Borrelia spirochetes. Light microscopy of a Giemsa-stained peripheral blood film of a 29 y/o male patient reveals multiple extracellular, helical spirochetes consistent with Borrelia species. The organisms appear as long, thin, undulating filaments with characteristic corkscrew morphology, distributed among red blood cells

The laboratory results revealed leukocytosis (13.26 × 10³/µL), severe thrombocytopenia (28,000/µL), AKI (creatinine of 1.42 mg/dL), and positive Borrelia spirochetes, and was negative for Plasmodium species on the blood film microscopy (Fig. 1 below).

Coagulation studies ruled out DIC with a prothrombin time (PT) of 15.1 s, an international normalization ratio (INR) of 1.15, and a partial thromboplastin time (PTT) of 30.5 s, as shown in Table 1 below.

**Table 1:** Laboratory trends on admission vs. Discharge of unusual presentation of borrelia associated relapsing fever with spontaneous hemoperitoneum

Laboratory parameter	On admission	At discharge	Reference range
Total White Cell Count	**13.26 × 10³/µL**	8.2 × 10³/µL	4–11 × 10³/µL
Neutrophil Count	88.3%	83.6%	40–70%
Platelet Count	**28**,**000/µL**	447,000/µL	150,000–450,000/µL
Hemoglobin	13.7 g/dL	**11.9 g/dL**	13.5–17.5 g/dL (Male)
Creatinine	**1.42 mg/dL**	0.5 mg/dL	0.7–1.3 mg/dL
Blood Urea Nitrogen (Urea)	**44.2 mg/dL**	30 mg/dL	7–20 mg/dL
Prothrombin Time (PT)	**15.1 s**	14 s	11–13.5 s
Activated PTT (PTT)	30.5 s	29 s	25–35 s
International Normalized Ratio (INR)	**1.15**	1.0	0.9–1.1
Alanine Aminotransferase (ALT)	**80 U/L**	47 U/L	7–55 U/L
Aspartate Aminotransferase (AST)	**66 U/L**	34 U/L	8–48 U/L
Amylase	68 U/L	38 U/L	30–110 U/L
Lipase	38 U/L	28 U/L	13–60 U/L
Blood Film	**Positive for Borrelia**	Negative	Negative

Transaminases (AST 66, ALT 80) suggested systemic inflammation. Resuscitation continued with crystalloids, oxygen, vasopressors, and hydrocortisone 50 mg IV QID, and doxycycline was given in addition to IV antibiotics, though blood culture was not sent as per the sepsis protocol.

Despite aggressive resuscitation, the patient developed worsening respiratory distress (SpO₂ of 79% at 15 L/min NRB, RR of 40/min, and accessory muscle use) and a declining GCS. The patient was subsequently intubated and put on mechanical ventilation (MV) via lung-protective ventilation with a tidal volume (Vt) of 420 mL, positive end expiratory pressure (PEEP) of 5 cmH_2_O, and a fraction of inspired oxygen (FiO₂) of 60%. The patient was sedated with ketamine infusion, and her SpO₂ improved to 99%.

In the absence of arterial blood gas analysis, ARDS was diagnosed clinically via bedside ultrasound (diffuse B-lines, consolidations), and severe hypoxemia was detected.

**Fig. 2:**
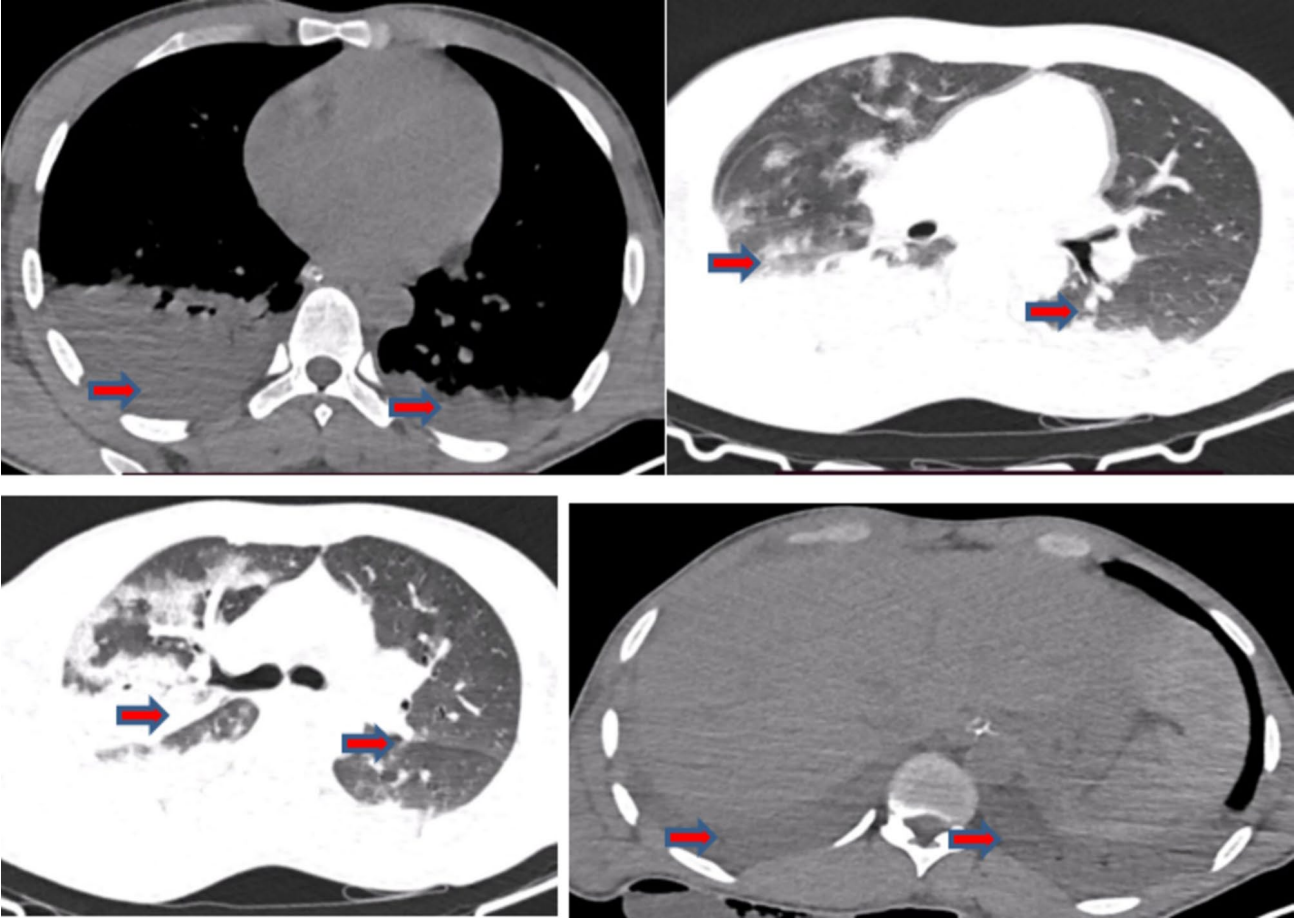
Chest CT scan revealed bilateral pleural effusions (right > left), widespread ground glass opacities (GGOs) with a nonhomogeneous distribution and a ventro-dorsal density gradient, with more dense consolidations in the dependent regions and a homogenous hypoechoic fluid collection (hemoperitoneum) in the peritoneum

A chest CT scan revealed bilateral pleural effusions, nonhomogeneous ground glass opacities, and a ventro-dorsal gradient of density, with dense consolidations in dependent regions and homogenous hypoechoic fluid collection in the peritoneum, as shown in Fig. 2 below.

The patient was transferred to the intensive care unit (ICU) with a working diagnosis of complicated relapsing fever with severe thrombocytopenia, RF-associated hemoperitoneum, acute kidney injury, and acute respiratory failure secondary to ARDS.

He received **doxycycline** 100 mg PO BID for 14 days, alongside empiric antibiotics of **cefepime** 1 gm IV TID and **vancomycin** 1 gm IV BID for 7 days, vasopressors, corticosteroids, mechanical ventilator support, and other critical care managements in the ICU. He stayed in the ICU for five days, and his condition improved gradually. He was extubated on the fourth day after ICU admission, and the vasopressors were discontinued.

He was transferred to the medical ward, and after a three-day stay, he was discharged. This case underscores **relapsing fever’s potential for multiorgan failure** (ARDS, hemoperitoneum, AKI) and the utility of bedside ultrasound in resource-limited settings.

## Discussion

In endemic regions such as Ethiopia, hemorrhagic peritoneal involvement has not been previously described in the medical literature despite extensive documentation of other bleeding complications [8, 9].

Our case establishes three important clinical imperatives:

RF must now be considered in the differential diagnosis of spontaneous hemoperitoneum in endemic areas. Bedside blood film microscopy remains the fastest and most reliable diagnostic tool in resource-limited settings [1, 4]., and capillary leak-mediated hemorrhage should be managed medically when no evidence of mechanical vascular injury exists [4, 10].

Relapsing fever (RF) causes hemoperitoneum through two mechanisms: Borrelia lipoproteins activate monocytes, leading to DIC and severe thrombocytopenia, and CXCL10-mediated endothelial injury, causing capillary leakage and hemorrhagic fluid buildup without physical rupture [1]. 

These processes explain why the patient *experienced* significant abdominal bleeding without the localized pain typical of surgical emergencies *such as* typhoid perforation [10].

Ethiopian RF outbreaks often cause bleeding complications, with 85% of patients experiencing thrombocytopenia and 32% showing mucosal bleeding, which are key signs of DIC when laboratory tests are unavailable [11].

There was an 18-year-old Somalian refugee with Borrelia recurrentis infection characterized by fever, hemoptysis, thrombocytopenia, and prolonged activated partial thromboplastin time, indicative of hemorrhagic diathesis, but no report of intra-abdominal bleeding with acute surgical abdomen [12].

Bedside ultrasound is used in Ethiopia to differentiate between surgical emergencies such as splenic rupture and RF-related hemoperitoneum, which is a common issue in low-resource settings [4, 10].

The patient’s hemoperitoneum was believed to be caused by a capillary leak rather than a ruptured blood vessel, resulting in normal hematocrit and the absence of focal peritonitis symptoms. Similar Borrelia infections in Ethiopia show similar patterns [4]

Unlike surgical emergencies (e.g., typhoid fever-related perforation or spleen rupture, which cause sudden pain and a decrease in hematocrit [10]), our case report does not require surgery.

In clinical practice, Giemsa-stained blood smear microscopy is used as the diagnostic test for acute relapsing fever, with greater than 90% sensitivity during febrile episodes [13].

Bakhtiari et al. (2004) validated the application of the clinical DIC criteria for coagulopathy assessment, which includes thrombocytopenia and bleeding manifestations [7, 14].

While the 20-minute whole blood clotting test (20WBCT) is recommended as a bedside tool for resource-limited settings, [6] it was not utilized in this case. Nonetheless, the combination of thrombocytopenia and clinical bleeding provides sufficient evidence to guide management. Rapid antibiotic therapy with cephalosporins or penicillin significantly reduces the spirochetal burden within 6–12 h, reversing coagulopathy and endothelial dysfunction in patients with severe relapsing fever [15]. This rapid bactericidal effect explains our patient’s ability to stabilize without surgical intervention.

## Conclusion and recommendations

A case of RF-associated hemoperitoneum highlights the importance of considering infectious causes and using bedside ultrasound, Giemsa smear, and immediate antibiotics to prevent unnecessary surgical intervention. Laparotomy poses risks, as it exacerbates capillary leak syndrome and may worsen intravascular coagulation. Clinicians in RF-endemic regions should consider RF in all patients with hemorrhagic shock and thrombocytopenia, prioritize rapid diagnostics, and limit surgical intervention to patients with definitive evidence.

## Limitations

This case report, despite limitations such as a lack of PCR confirmation, the potential influence of empiric antibiotic administration, and a lack of advanced coagulation tests, highlights the potential of conservative management for severe RF complications.

## Data Availability

This information will be provided upon request from the corresponding author without limitations.
